# A cluster randomised controlled trial of the *Climate Schools: Ecstasy and Emerging Drugs Module* in Australian secondary schools: study protocol

**DOI:** 10.1186/1471-2458-13-1168

**Published:** 2013-12-12

**Authors:** Katrina E Champion, Maree Teesson, Nicola C Newton

**Affiliations:** 1 NHMRC Centre for Research Excellence in Mental Health and Substance Use, National Drug and Alcohol Research Centre, University of New South Wales, 22-32 King Street, Randwick 2052, NSW, Australia

**Keywords:** Internet, Prevention, Universal, School, Ecstasy, New and emerging drugs, Substance use

## Abstract

**Background:**

The use of ecstasy is a public health problem and is associated with a range of social costs and harms. In recent years, there has been growing concern about the availability and misuse of new and emerging drugs designed to mimic the effects of illicit drugs, including ecstasy. This, coupled with the fact that the age of use and the risk factors for using ecstasy and emerging drugs are similar, provides a compelling argument to implement prevention for these substances simultaneously. The proposed study will evaluate whether a universal Internet-based prevention program, known as the *Climate Schools: Ecstasy and Emerging Drugs Module,* can address and prevent the use of ecstasy and emerging drugs among adolescents.

**Methods:**

A cluster randomised controlled trial will be conducted among Year 10 students (aged 15–16 years) from 12 secondary schools in Sydney, Australia. Schools will be randomly assigned to either the *Climate Schools* intervention group or the control group. All students will complete a self-report questionnaire at baseline, immediately post-intervention, and 6-, 12- and 24-months post-baseline. The primary outcome measures will include ecstasy and emerging drug-related knowledge, intentions to use these substances in the future, and the patterns of use of ecstasy and emerging drugs. A range of secondary outcomes will also be assessed, including beliefs and attitudes about ecstasy and emerging drugs, peer pressure resistance, other substance use and mental health outcomes.

**Discussion:**

To our knowledge, this will be the first evaluation of an Internet-based program designed to specifically target ecstasy and NED use among adolescents. If deemed effective, the *Climate Schools: Ecstasy and Emerging Drugs Module* will provide schools with an interactive and novel prevention program for ecstasy and emerging drugs that can be readily implemented by teachers.

**Trial registration:**

This trial is registered with the Australian New Zealand Clinical Trials Registry, ACTRN12613000708752.

## Background

The use of illicit drugs among Australians is a significant public health problem and is associated with considerable social costs and harms [1]. Use among young Australians is especially concerning, with nearly one-quarter of teenagers aged between 14–19 years reporting having tried an illicit drug in their lifetime [2]. In 2010, ecstasy was the second most commonly used illicit drug in Australia after cannabis. Although the prevalence of ecstasy use in adolescents is relatively low, with less than three per cent of 14–19 year olds reporting any use in the past 12 months [2], for those teenagers who do use ecstasy, the potential for harm is considerable. Ecstasy use has been associated with a range of serious adverse effects including an increased likelihood of having a mental illness and greater levels of psychological distress [2]. Furthermore, early initiation to substance use is a risk factor for a range of negative consequences including using other drugs in adulthood, juvenile offending, poor academic performance, delinquency and school dropout [3-6].

In recent times, concern has mounted about the manufacture and misuse of New and Emerging Drugs (NEDs), a general term used to refer to substances that are not under international control [7]. Also known as ‘emerging psychoactive substances’, ‘legal highs’ and ‘synthetic drugs’, NEDs are specifically designed to mimic the effects of existing illicit substances, such as ecstasy, by slightly changing their chemical structure [8]. The term NEDs includes a wide range of synthetic substances, however there are two common types: *synthetic cathinones*, stimulant-like substances intended to imitate ecstasy and amphetamine, and *synthetic cannabinoids,* compounds designed to mimic the effects of cannabis [8-10]. Statistics from the United States indicate that in 2012, less than one per cent of American tenth-graders (aged 15 years) had used a synthetic cathinone in the past year, but almost nine per cent had used synthetic marijuana [11]. In addition, a recent United Nations report indicated that adolescents as young as 15 years old are using NEDs in the European Union (EU) [7]. Although there is no available data on the prevalence or age of NED use among young Australians, internet monitoring analyses indicate that there are a large number of emerging substances available to Australian consumers through online retailers [12,13]. Indeed, the rate at which these drugs are emerging is alarming, with 73 new psychoactive substances notified for the first time in the EU in 2012 [14]. This rapid growth and availability of NEDs, at both a global and local level, and the likely harms associated with their use, are a potential cause for concern.

Although NEDs are manufactured to imitate the effects of existing substances, the use of NEDs is associated with a number of unique risks and challenges compared to established illicit drugs. Firstly, since these substances are emerging so rapidly and are constantly changing, very little data exist on their toxicology and the risks associated with their use [15]. Short-term side effects reported by users include agitation, psychosis, insomnia, palpitations and nausea [10,16], however there is a complete lack of knowledge about the effects of NEDs in the long term. Further compounding this problem is the huge array of NEDs that are available, all of which are likely to have different effects and different risk profiles [17]. Secondly, the fact that these substances are often marketed as ‘legal highs’, ‘bath salts’ or ‘plant food’ is likely to influence people’s perceptions of the risks associated with their use [18]. That is, young people are led to incorrectly believe that these substances are low-risk and safe to use, despite there being no evidence to support this. In light of the uncertainty about the adverse effects of NEDs, and the huge potential for young people to misuse these substances, the United Nations has urged governments to educate adolescents about NEDs through drug prevention programs [7,9]. Therefore, there is a clear need to respond to this new public health challenge with the development of evidence-based prevention programs for NEDs.

Given the overlap in the age of use, risk factors and potential harms associated with ecstasy and NED use [2,7], as well as the fact that NEDs are often produced to imitate the psychoactive effects of ecstasy, it is logical to deliver prevention for these substances simultaneously. School is the ideal location to implement such prevention, as young people spend over a quarter of their waking lives at school [19] and in many States in Australia, delivering drug education at school is mandatory. Despite the existence of school-based prevention programs, their efficacy has been limited, especially their ability to change behaviour and reduce substance use [20,21]. This is likely due to obstacles that impede program implementation, such as a lack of resources in terms of teachers, time and money available [22], as well as the fact that teachers often make unfavourable adaptations to program content [23,24]. Internet-based programs appear to overcome these barriers and offer greater accessibility, affordability, and feasibility of use compared to traditional programs [25,26]. Despite these advantages, few Internet-based prevention programs have been developed for illicit drugs, with most focussing on alcohol and tobacco use [27], and there are no existing Internet-based programs specifically targeting ecstasy misuse and the growing phenomenon and use of NEDs. In response to this, the aim of the current study is to build on the successful *Climate Schools* framework to develop and evaluate an online, school-based prevention program solely for ecstasy and NEDs.

### The Climate Schools framework

The *Climate Schools* courses are school-based prevention programs for alcohol and other drugs, based on a harm minimisation approach and social learning principles. The *Climate Schools* courses are delivered via the Internet, and engage students through online cartoon storylines. A number of *Climate Schools* courses have previously been trialled among Australian school students, with results supporting their feasibility and efficacy in reducing harmful alcohol and cannabis use [28-31]. Specifically, the *Climate Schools: Alcohol Module* has been evaluated with two separate cluster randomised controlled trials (RCTs) and has been found to increase alcohol-related knowledge, decrease positive expectancies about alcohol and to reduce average alcohol consumption, the frequency of binge drinking and alcohol-related harms among Australian Year 8 students [30,31]. Furthermore, a recent cluster RCT (n = 764 students) of the *Climate Schools: Alcohol and Cannabis* course in 10 Sydney secondary schools [28,29] was also successful in increasing cannabis and alcohol-related knowledge, and decreasing the average consumption of alcohol use and the frequency of cannabis use and binge drinking amongst young people. It is also important to note that both teachers and students rated the *Climate Schools* programs as an enjoyable, useful and relevant drug education resource. Therefore, these results position the *Climate Schools* platform as a sound foundation upon which to base a new prevention program that specifically addresses ecstasy and NEDs.

### Aims and hypotheses

The aim of the current study is to determine whether the successful *Climate Schools* framework can be extended to the prevention of established illicit drugs, such as ecstasy, as well as emerging drugs. To our knowledge, this will be the first trial of an Internet-based prevention program targeting ecstasy and NED use among young people. Specifically, the proposed study will seek to determine whether the *Climate Schools: Ecstasy and Emerging Drugs Module* is more effective than school-based health education as usual in:

1) Increasing ecstasy- and NED-related knowledge

2) Reducing intentions to use ecstasy and NEDs

3) Preventing the uptake and reducing the use of ecstasy and NEDs

Secondary aims include examining the effects of the intervention on ecstasy- and NED-related beliefs and attitudes, peer pressure resistance, mental health outcomes, other substance use and truancy.

## Methods

### Developing the *Climate Schools: Ecstasy and Emerging Drugs Module*

In 2009 the Australian Government Department of Health and Ageing (DoHA) commissioned a study to develop the *Climate Schools: Ecstasy Module*. The initial development of this program resulted in three Internet-based lessons which aimed to educate adolescents about the harms associated with ecstasy use [11]. In 2013 the *Climate Schools: Ecstasy Module* was modified to ensure that the existing content was up-to-date and relevant for teenagers today, and to incorporate new content about NEDs and associated harms. To achieve this, an additional cartoon lesson was added to the module, resulting in a four-lesson prevention program for ecstasy and NEDs called the *Climate Schools: Ecstasy and Emerging Drugs Module.* Focus testing was conducted with students (n = 7), who were asked to provide feedback about the language used in the cartoon storyline and on the relevance and acceptability of the program to people their age. Health professionals (n = 6) in the field of drug and alcohol research were also asked to review the language and content of the cartoon script. Student activities and teacher resources were updated to reflect the new lesson content about NEDs and to include the most recent prevalence data from Australia [32]. Furthermore, based on teacher feedback from previous trials [33], new Internet-based student activities were created to increase the level of interactivity and to maximise student engagement and learning. This focus on interactive student learning is reflected in the name of current study, which is known as the *Climate Schools Interactive* (CSI) Study.

### Study design

A cluster RCT will be conducted in 12 secondary schools in the greater Sydney region between 2014 and 2016. Six schools will be randomly assigned to the C*limate Schools* intervention group, and six schools to the control group. Cluster randomisation will be employed in this study to avoid contamination of the control group by the intervention group through student communication [34]. To evaluate the *Climate Schools* intervention for efficacy, students in both groups will complete five online self-report questionnaires over the three year study period. The trial has been approved by the University of New South Wales Human Research Ethics Committee (HREC HC13075).

### Sample size calculations

To account for cluster randomisation, sample size calculations are based on recent sample size requirements developed by Heo and Leon [35] to detect intervention by time interactions in longitudinal cluster randomized clinical trials. To detect differences between groups, five schools (with an average of 75 students each) will need to be randomly allocated to receive the *Climate Schools* intervention, and five schools to a control group. Based on a recent school-based trial conducted by the investigators [33], in which there was an average of 125 students per grade in Sydney secondary schools, recruiting 75 students per school appears feasible. This will achieve 80 per cent power to detect a standardized between-group mean difference of 0.20 (p = 0.05) in outcomes at the end of the trial with three measurement occasions. An effect size of 0.20 for use is comparable to previous trials of universal drug prevention programs [19,27]. To account for school dropouts during the trial, which we expect to be approximately 10 per cent [28], we aim to recruit at least 12 schools, providing a minimum of 900 students at baseline to test the effect of the intervention in the overall group.

### Recruitment and randomisation of schools

**Figure 1:**
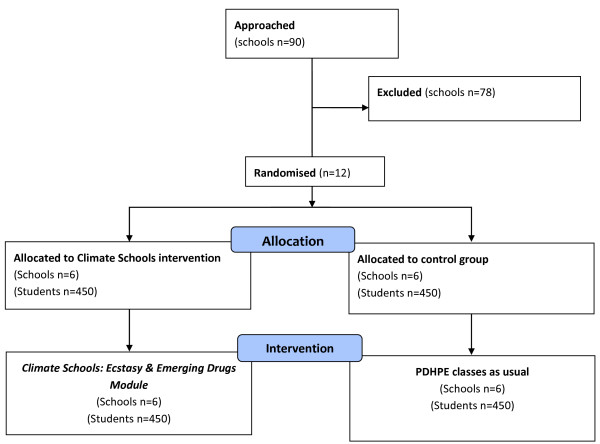
Anticipated recruitment and allocation of schools.

Approximately 90 Independent schools in the greater Sydney region will be invited to participate in the trial. Schools that have previously collaborated with the researchers or that have expressed an interest in participating in future research will be approached initially. School principals will be sent a letter outlining the aims of the study and seeking their permission to conduct research with their students. Principals that do not respond to the initial mail-out will receive a further email and phone call from the researchers to determine if they are interested in participating in the study. Following school consent, randomisation will occur using the RALLOC function in Stata, and schools will be randomly assigned to either the *Climate Schools* group or the control group. Neither teachers, parents nor students will blinded to school randomisation, however research staff administering student surveys will be kept blind to the allocation of schools. Based on the sample size calculations described previously, 12 schools, with approximately 900 male and female students will be recruited and randomised (see Figure 1). Students will be in Year 10, aged between 15–16 years, at baseline and will be in Year 12, aged between 17–18 years, at the final follow-up occasion. Participating schools will be asked to distribute Information and Consent Forms to all Year 10 parents at the start of the school year in 2014. Schools have the option of sending the forms home with students, mailing them to parents or emailing them to parents. In line with other school-based studies currently being undertaken by the investigators, passive parental consent will be employed. This is based on research indicating that active consent procedures can result in the exclusion of certain demographic and high-risk groups, having the potential to introduce a degree of selection bias into studies of adolescents’ substance use [36,37]. It can also dramatically reduce participation rates [33]. Furthermore, teacher feedback from a previous trial run by the investigators [33] also indicates that active consent can exclude certain types of students and that passive parental consent is the preferred procedure by many schools. In addition to the Parental Information and Consent forms, school principals will be asked to send a letter directly to Year 10 parents (via mail or email) with information about the project and how they can withdraw their child from participating. Students will also be required to provide active consent themselves to be eligible for the study.

### The Climate Schools intervention

Schools randomly allocated to the *Climate Schools* intervention will be asked to implement the *Climate Schools: Ecstasy and Emerging Drugs Module* with all Year 10 students during Personal Development, Health and Physical Education (PDHPE) classes. Prior to the implementation of the intervention, the researchers will meet with Year 10 PDHPE teachers at participating schools to brief them about the *Climate Schools* program, reiterate the study aims and assist them in navigating the study website. The *Climate Schools: Ecstasy and Emerging Drugs Module* consists of four 40-minute lessons, which are intended to be delivered once weekly over a four-week period. Each lesson consists of a 20-minute online cartoon component, completed individually by the student, followed by 20-minutes of online and teacher-delivered class activities. The four lessons cover content about what ecstasy and NEDs are, consequences of use, drug refusal skills and strategies for staying safe (see Table 1 for a full outline of the lesson content). The program was developed to address learning outcomes from the NSW Stage 5 PDHPE syllabus, and will be realigned with the new Australian Health and Physical Education curriculum once released. Students and teachers will access the *Climate Schools* cartoons and online activities via the study website (http://www.csistudy.org.au). Teachers will also be provided with online access to implementation guidelines, links to the education syllabus and summaries for each lesson.

**Table 1:** **Lesson content of the *climate schools: ecstasy and emerging drugs module***

**Lesson**	**Content**
1	• What are new and emerging drugs?
	• What is ecstasy?
	• Emerging drugs and legal issues
	- ‘Legal Highs’: not necessarily legal
	- Legal does not mean safe
	• Impact of ecstasy on the body
	• Consequences of emerging drug use
	• Prevalence of ecstasy use among teenagers
	• Acceptability of emerging drug use among peers
2	• Negative impacts of ecstasy and emerging drugs on relationships
	• Risk taking behaviour and consequences
	• Keeping safe
	• Mixing ecstasy and emerging drugs with alcohol
	• Acute/short term effects of ecstasy and emerging drugs
	• The ‘come down’ from ecstasy and emerging drugs
	• The unknown: What do emerging drugs contain and what will they do?
3	• Social implications of ecstasy and emerging drug use
	• Health issues associated with ecstasy and emerging drug use
	• Consequences of risk taking behaviour
	• Financial implications of ecstasy and emerging drug use
	• Unpleasant psychological effects of ecstasy and emerging drug use
	• What do pills really contain?
	• Poly drug use – dangers of mixing pills with alcohol and other drugs
	• Saying no to drugs – effective communication skills
4	• Health and safety issues associated with ecstasy and emerging drug use
	• Drug-related emergencies
	• Harm minimisation strategies
	• Poly drug use revisited
	• Saying no to drugs - effective communication skills revisited
	• Ecstasy, emerging drugs and the law

### The control group

Students attending schools allocated to the control group will receive their standard PDHPE lessons (which cover drug education topics) over the course of 2014. At the end of the year, teachers at control schools will be asked to complete a brief survey that asks about the amount and format of any drug education they delivered to their Year 10 students. Control schools will be offered complimentary use of the *Climate Schools: Ecstasy and Emerging Drugs Module* at the end of the study period.

### Assessment

Students in both groups will complete an online self-report questionnaire at baseline, immediately post-intervention and 6-, 12- and 24-months later. Students will be required to create a unique username and password to register with the study website and will use these details to login to complete each survey. Upon registration participants will be automatically assigned a unique code, which will be used to link student data across the survey time points whilst maintaining confidentiality. Table 2 outlines the assessment and intervention timeline for both groups.

**Table 2:** Assessment and intervention timeline

	**Baseline**	**Climate Schools program**	**Immediate Post-test**	**6 month F/U**	**12 month F/U**	**24 month F/U**
	**Survey**					
**Time**	Term 1	Term 1	Term 1	Term 3	Term 1	Term 1
	2014	2014	2014	2014	2015	2016
**Grade**	Year 10	Year 10	Year 10	Year 10	Year 11	Year 12
**Age**	15-16yrs	15-16yrs	15-16yrs	15-16yrs	16-17yrs	17-18yrs
CO*	✓	✗	✓	✓	✓	✓
CL*	✓	✓	✓	✓	✓	✓

*CO = Control group, CL = Climate Schools Intervention group.

**Figure 2:**
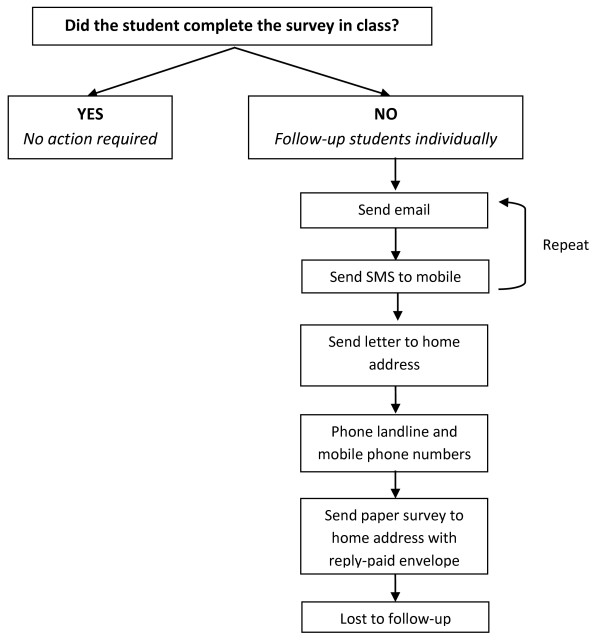
Follow-up procedure for student surveys.

Students will complete the 15-minute survey during class time, as directed by their teacher. Students that are absent on the day of the survey occasion will be contacted directly by the researchers, using contact details provided by students when consenting and registering online. Students will be contacted to complete the survey using the procedure outlined in Figure 2 below. This procedure has been employed by the investigators in the past [33] and was found to be successful in increasing survey retention rates at follow-up.

### Outcomes

Demographic data including gender, age, religion, academic performance and truancy rates will be obtained to determine baseline equivalence of the groups.

### Knowledge about ecstasy and NEDs

Knowledge about ecstasy and NEDs will be measured using a 20-item scale specifically developed to reflect the intended content of the *Climate Schools* intervention. Items cover knowledge of ecstasy and NEDs in relation to the drugs themselves, prevalence of use, physical and mental effects, legal consequences and information to minimise the harm associated with their use. For each of the 20 statements, students are required to answer ‘True’, ‘False’ or ‘Don’t Know.

### Intentions to use ecstasy and NEDs

Intentions to use ecstasy, synthetic cannabis, synthetic stimulants (‘bath salts’) and NEDs in general will be assessed using eight items. Students will be asked to rate how likely they are to use each of these substances in the ‘next six months’ and ‘at any time in the future’. Each item requires students to rate their intention on a five-point Likert scale labelled ‘very likely’ to ‘very unlikely’.

### Patterns of ecstasy and NED use

Patterns of ecstasy and NED use will be assessed using questions adapted from the 2010 National Drug Strategy Household Survey (NDSHS) [2]. Students will be asked if they have ever used ecstasy or an emerging drug (either synthetic cannabis or synthetic stimulants/‘bath salts’), the age of first use, use in the past six months, use in the past month as well as the frequency and quantity of use. The distinction between synthetic cannabis and synthetic cathinones is consistent with the *Monitoring the Future* survey from the United States [11] and will allow for international comparison of student prevalence data.

### Other substance Use

Students will be asked to rate the frequency and quantity of their alcohol use in the past six months and the frequency of drinking to excess (consuming five or more standard drinks on one occasion). Other substance use will be measured using four items that ask whether students have tried tobacco, cannabis, methamphetamine/amphetamine or any other substance in the past six months. Possible responses are ‘never’, ‘tried once’, ‘tried more than once and less than five times’ or ‘tried five times or more’.

### Beliefs about the consequences of ecstasy and NED use

Beliefs about the consequences of ecstasy and NED use will be measured using items adapted from the Project ALERT questionnaire [38]. To elicit perceptions about the social consequences of using ecstasy and NEDs, students will be asked about the positive and negative expectancies of using these drugs, for example, *‘using ecstasy and emerging drugs makes you feel more confident’.* Responses will be made on a four-point Likert scale labelled ‘strongly agree’, ‘sort of agree’, ‘sort of disagree’ and ‘strongly disagree’.

### Normative beliefs

To measure normative beliefs, students will be asked to estimate the proportion of their peers that use ecstasy and NEDs. Participants will also be asked three items adapted from the Project ALERT questionnaire [38], which aim to measure peer tolerance about ecstasy and NED use. For example, students will be asked to anticipate their friends’ reactions if they *‘found out you used ecstasy or emerging drugs sometimes’* (1 = ‘they would disapprove and stop being my friends’ to 4 = ‘they would approve’). Students will also be asked to indicate how strongly they agree or disagree with three statements relating to perceptions of the legality and safety of NEDs.

### Mental health outcomes

Psychological distress will be measured using the *Kessler 6* scale [39], a six-item questionnaire that measures depressive and anxiety symptoms in the past four weeks. The 25-item *Strengths and Difficulties Questionnaire*[5] will be used to measure both positive and negative attributes of students.

### Peer pressure

Peer pressure resistance will be measured using Bandura’s *Resistive Self-regulatory Efficacy scale*[40]. This scale consists of nine items that ask students to rate how well they can resist peer pressure to engage in different behaviours e.g. ‘*How well can you resist peer pressure to use pills (ecstasy)’.* Responses are made on a seven-point scale ranging from ‘Not well at all’ to ‘Very Well’.

### Parental monitoring

Students will be asked *‘Do your parents know where you are if you go out in the evening?’ and ‘Do your parents know whom you meet if you go out in the evening?’* and to make a response on a five-point scale ranging from ‘Yes, always’ to ‘No, never’. These items are commonly combined to provide an indicator of parental monitoring [41].

### Peer deviance

Five items adapted from a study by Svensson [41] will be used to measure relations with deviant peers. Students will be asked whether they have a friend who engages in deviant behaviour, for example, ‘*do you have a friend who has stolen something from a store?’ ,* and to make a Yes/No response.

### Program evaluation and implementation fidelity

Students that receive the *Climate Schools* intervention will be asked to complete an online evaluation questionnaire at the end of the final cartoon lesson. Teachers will also be asked to complete an online questionnaire at the conclusion of the program. To monitor adherence to the intervention, teachers will be required to complete a fidelity logbook at the end of each lesson via an online survey. The logbooks ask teachers to indicate which lessons and activities they completed with their class and to write down any factors that may have disrupted or impeded delivery of program. To ensure complete and consistent delivery of the online component of the intervention, the study website has been programmed so that students will be required to view the *Climate Schools* lessons in full before being granted access to the following lesson.

### Statistical analysis

Single-level analyses; one-way analyses of variance (for normally distributed data), Chi-square (for binominal data), and Mann–Whitney U-test (for non-normally distributed data) will be used to examine baseline equivalence and attrition between groups. Due to the multi-level and hierarchical nature of the data, mixed effects regression will be used to examine intervention by time interaction effects. To account for intracluster variance within schools, intervention effects will primarily be examined using hierarchical linear modelling (HLM) for normally distributed data and hierarchical generalized linear modelling using a Poisson distribution for count data. Outcome variables will be centred at post-test to allow for comparisons between groups immediately after the intervention, and growth terms will be analysed to determine the magnitude of the follow-up effects. Analyses will be conducted using the program Stata. If unconditional models reveal that less than 10% of systematic variance exists between school level for any outcome variable, HLM will be abandoned and single-level analyses will be used [4]. For these variables, ANCOVAs utilising the SPSS GLM procedure will be conducted to account for any baseline differences that might exist between groups. For multiple comparisons Bonferroni adjustments will be made. Effect sizes, odds ratios and 95% confidence intervals will also be calculated.

## Discussion

The aim of the current study is to implement and evaluate the *Climate Schools: Ecstasy and Emerging Drugs Module* through a cluster RCT among Australian students*.* It is hypothesised that the *Climate Schools* intervention will increase ecstasy and NED-related knowledge, reduce intentions to use ecstasy and NEDs in the future and prevent the uptake and reduce the use of ecstasy and NEDs. Given the considerable attention NEDs have received by policymakers, researchers and the media recently, a clear strength of the proposed study is that it is a timely intervention. The evaluation of the *Climate Schools: Ecstasy and Emerging Drugs Module* addresses the calls to action raised by major international bodies, such as the United Nations, to develop and implement universal prevention programs for NEDs among young people [9]. To our knowledge, this will be the first trial of an online, school-based prevention program designed to specifically target ecstasy and NED use among adolescents.

It is anticipated that the *Climate Schools: Ecstasy and Emerging Drugs Module* will build on the success of the existing *Climate Schools* courses [28-31], and provide schools with an interactive, affordable and evidence-based prevention program for ecstasy and NEDs that can be readily implemented by teachers. A further strength of the proposed study is the simultaneous delivery of education for ecstasy and NEDs. Due to the overlap between the substances, that is, NEDs are often manufactured to produce ecstasy-like psychoactive effects, they are both often available in pill form, and the age of use for both substances are similar, it is logical to integrate prevention for ecstasy and NEDs. By delivering one four-lesson program for both ecstasy and NEDs, teachers are able to educate students about these drugs in a time-effective manner, whilst maximising prevention messages. Furthermore, through its online delivery, the *Climate Schools* intervention is likely to be appealing to teenagers and foster interactive learning and high student engagement. Of most significance, if the *Climate Schools: Ecstasy and Emerging Drugs Module* can prevent the uptake and reduce the use of ecstasy and NEDs, it is possible that the program can minimise the potential burden of disease, social costs, and disability associated with their misuse.

## Competing interests

NN, MT and KC are three of the developers on the *Climate Schools: Ecstasy and Emerging Drugs Module.* This program is distributed not for profit. The other authors declare that they have no competing interests.

## Authors’ contributions

KC, MT and NN were involved in the study design and are responsible for the ethics and clinical trial submission, recruitment of schools and data collection. KC, MT and NN contributed to the manuscript preparation and read and approved the final manuscript.

## Pre-publication history

The pre-publication history for this paper can be accessed here:

http://www.biomedcentral.com/1471-2458/13/1168/prepub
